# Diarrhea no more: does zinc help the poor? Evidence on the effectiveness of programmatic efforts to reach poorest in delivering zinc and ORS at scale in UP and Gujarat, India

**DOI:** 10.7189/jogh.06.021001

**Published:** 2016-12

**Authors:** Amnesty E LeFevre, Diwakar Mohan, Sarmila Mazumder, Laura L. Lamberti, Sunita Taneja, Robert E Black, Christa L Fischer–Walker

**Affiliations:** 1 Department of International Health, Johns Hopkins Bloomberg School of Public Health, Baltimore, MD, USA; 2 Centre for Health Research and Development, Society for Applied Studies, New Delhi, India

## Abstract

**Background:**

India has the greatest burden of diarrhea in children under 5 years globally. The Diarrhea Alleviation through zinc and oral rehydration salts (ORS) Therapy program (2010–2014) sought to improve access to and utilization of zinc and ORS among children 2–59 months in Gujarat and Uttar Pradesh (UP), India, through public and private sector delivery channels. In this analysis, we present findings on program’s effect in reducing child–health inequities.

**Methods:**

Data from cross–sectional baseline and endline surveys were used to assess disparities in key outcomes across six dimensions: socioeconomic strata, gender, caregiver education, ethnicity and geography.

**Results:**

Careseeking outside the home for children under 5 years with diarrhea did not increase significantly in UP or Gujarat across socioeconomic strata. Declines in private sector careseeking were observed in both sites along with concurrent increases in public sector careseeking. Zinc, ORS, zinc+ORS use did not increase significantly in UP across socioeconomic strata. In Gujarat, increases in zinc use (20% overall; 33% in the Quintile 5 (Q5) strata) and zinc+ORS (18% overall; 30% in the Q5 strata) were disproportionately observed in the high income strata, among members of the most advantaged caste, and among children whose mothers had ≥1 year of schooling. ORS use increased significantly across all socioeconomic strata for children in Gujarat with diarrhea (23% overall; 33% in Q5 strata) and those with dehydration + diarrhea (33% overall; 38% in Q5 strata). The magnitude of increase in ORS receipt from the public sector was nearly twice that observed in the private sector. In Gujarat, while out of pocket spending for diarrhea was significantly higher for male children, overall costs to users declined by a mean of US$ 2; largely due to significant reductions in wages lost (–US$ 0.79; *P* < 0.003), and transportation costs (–US$ 0.44; *P* < 0.00).

**Conclusions:**

While significant improvements in diarrhea treatment were achieved in Gujarat, new strategies are needed in UP, particularly in the private sector. If national–level reductions in diarrheal disease burden are to be realized, improved understanding is needed of how to optimally increase coverage of zinc and ORS and decrease contraindicated treatments amongst the most disadvantaged across geographic areas and axes of gender, ethnicity, education and socioeconomic status.

Diarrhea is one of few infections, which underpins the gap in child survival between the world’s poorest and richest countries [1,2]. Despite declines of nearly 50% since 2000, an estimated 578 000 deaths in children under 5 in 2013 were attributed to diarrhea [3]. The majority of deaths due to diarrhea occur in low resource settings; one in four in India alone [3].

Diarrhea is a disease of the poor. Defined by the passing of three or more loose or liquid stools in 24 hours, diarrhea is caused by viruses, bacteria, and/or parasites spread through contaminated food, water, or person–to–person contact resulting from poor hygiene and inadequate sanitation. Vast differentials in sanitation coverage and access to safe water across population sub–groups underscore the differential risk of diarrheal diseases on the poorest children.

In nearly all diarrhea cases, death stems from fluid loss and dehydration. While oral rehydration solution (ORS) – effective in reducing morbidity and mortality due to dehydration – has been used as a mainstay of treatment diarrhea since 1978, its use is limited to only 38% of diarrheal episodes globally and 26% in India [4,5]. Overall use rates mask wide disparities across wealth groups, with only 19% of the poorest and poor in India receiving ORS as compared to 43% in the highest wealth groups [4]. Further disparities in treatment and health outcomes by age, sex, education, and geography have been reported for diarrhea management in Bangladesh [6] and other child health outcomes in India [7].

Zinc supplementation for the treatment of acute diarrhea has been shown to decrease the duration and severity of the diarrheal episodes, rates of hospitalization for diarrhea, and all–cause and diarrhea mortality [8]. As a result, in 2004 the World Health Organization (WHO) and UNICEF amended global guidelines for the management of acute diarrhea to include the recommendation that children receive zinc supplementation for 10–14 days, in addition to ORS and continued feeding [9]. Yet over a decade later, zinc availability remains limited and its use rates unknown, particularly amongst the poorest.

To address persistent gaps in diarrhea treatment in India, the Diarrhea Alleviation through zinc and ORS Therapy (DAZT) program was initiated in 2010 to improve access to and utilization of zinc and ORS among children 2–59 months in 6 of 26 districts in Gujarat State, India and 12 of 75 districts of Uttar Pradesh (UP), India. In this analysis, we present findings related to the effect of DAZT programmatic activities on equity in caregiver knowledge, careseeking, treatment, cost, and diarrheal disease prevalence following zinc introduction.

## METHODS

### Key definitions and terminology

**Figure 1:**
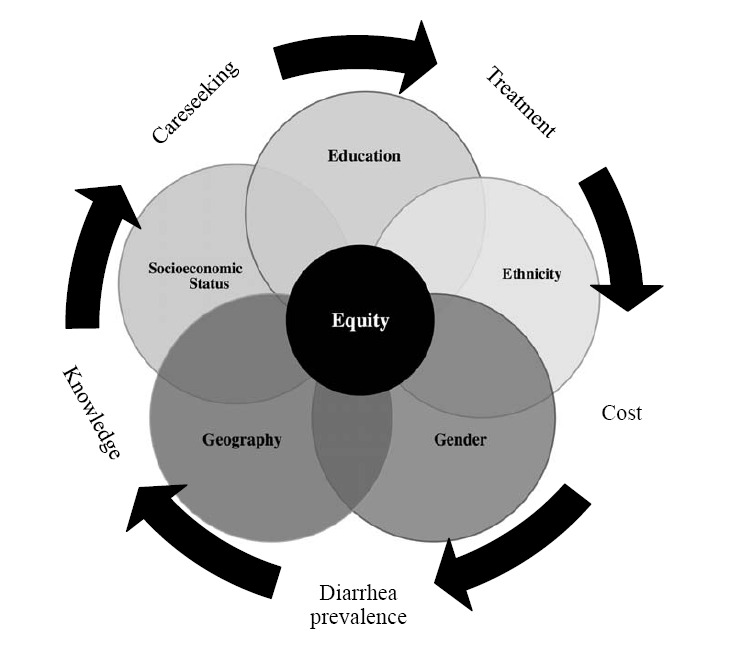
Framework for assessing the effects of multiple dimensions of equity influencing knowledge, careseeking, treatment, cost and health status for diarrhea.

In the context of the DAZT program, we sought to assess horizontal equity (treating like cases of diarrhea alike) by exploring the effects of program activities across population subgroups on key outcomes, including caregiver knowledge of zinc and ORS, careseeking overall and by sector, treatment, and diarrheal disease prevalence [10,11]. Vertical equity (treating unlike cases of diarrhea differently) was explored through analyses of different types of diarrhea (eg, diarrhea with and without dehydration) and the effects on ORS use across population subgroups. In contrast to many analyses, which focus on one dimension of equity defined by socioeconomic status and measured by wealth quintiles [12–14], we additionally considered the distributional impacts according to gender, education, ethnicity and geography at a sub–national level by comparing UP with Gujarat (**Figure 1**). Elsewhere the importance geography at a sub–national level [6] has been noted for diarrhea in Bangladesh. However, the constraints of DAZT program implementation to rural areas at sub–state level limited our inclusion of this added dimension.

### Study sites and context

Gujarat (population 60 million) and Uttar Pradesh (population 199 million) are states in West and Central India, respectively [15]. Project activities were implemented among a population of 41 million (6.3 million under 5 population) across 12 districts of UP and 13 million (2.1 million under 5 population) across 6 districts of Gujarat [15]. With a Gross Domestic Product (GDP) of US$ 2337, Gujarat ranks among India’s top 10 states for economic productivity. By comparison, with a GDP of US$ 793, UP is second only to Bihar as the worst performing state in India. Economic trends underscore disparities in disease burden, careseeking, and health practices across states. In UP, nearly 1–in–10 children die prior to reaching their fifth birthday; only 30% receive all 3 DTP immunizations, and 23% receive all basic immunizations [4,16]. In Gujarat, for every 1000 live births, 56 children die prior to their fifth birthday [4,16]. While immunization rates exceed those reported in UP, deficits remain with only 61% of children receiving all 3 DTP immunizations and 45% receiving all basic immunizations [4]. Prior to the implementation of DAZT, among children with diarrhea, 58% of children in UP and 57% in Gujarat were taken to a health care provider [4]. In Gujarat, 26% received ORS and 31% unknown drugs as compared to 13% and 47%, respectively, in UP [4].

### Program description

A detailed overview of the DAZT program has been published elsewhere [17]. Project activities sought to improve the availability of and access to ORS and zinc for the management of acute diarrhea through public and provider delivery channels in Gujarat and UP. Public sector activities were concentrated in primary health centers and among community based providers, including Accredited Social Health Activists (ASHAs) and Anganwadi workers (AWWs). Programmatic inputs included training in overall diarrhea prevention and management to district and block level supervisors, as well as facility and community based health providers. To address immediate shortages in supplies, the program provided an initial seed supply of diarrhea treatment kits (DTKs) comprised of two ORS sachets, 10 taste masked zinc tablets, a measuring cup, and an informational leaflet for caregivers.

Private sector activities included work with pharmaceutical companies, homeopathic and alternative medicine associations, to promote zinc and ORS use and ensure product supply among informal and formal private providers in both states. Implementation involved a push and pull strategy. The former sought to change prescription practices among key opinion leaders in the medical community and market ORS and zinc to private providers, while the latter, pull strategy sought to create demand for ORS and zinc amongst private providers. Programmatic inputs included the provision of Information Education and Communication (IEC) materials, and training in diarrhea prevention and management, including the importance of zinc and ORS, dosage and regulatory guidelines, as well as promotional strategies for effective product placement. DAZT corners were additionally established as informational booths in private clinics and hospitals to create awareness among caregivers and remind providers to prescribe zinc.

### Sampling and study design

The details of the study design and sampling plan are provided in great details elsewhere [18]. Briefly, the evaluation of the DAZT program assumed a prospective, pre–post design with activities primarily comprised of cross–sectional surveys conducted at baseline and endline (**Figure 1**). Surveys assessed household characteristics, caregiver knowledge, care seeking behavior, out of pocket expenditures, and household assets for classifying individuals according into wealth quintiles [18].

A post–hoc power analysis was performed to identify the lowest detectable difference between the wealth groups for the outcome of ORS use among respondents with diarrhea in the past 2 weeks. The analysis was based on a minimum sample size of 84 (least poor at endline for Gujarat) per group, type 1 error of 0.05, and power of 80%. For ORS use with baseline prevalence of 16%, the minimum detectable difference was 18% while for zinc use with baseline prevalence of 2%, the minimum detectable difference was 11%.

### Data analyses

All data analyses were conducted in Stata 13 (StataCorp, College Station, TX, USA). Bivariate analysis was used to analyze demographic and socioeconomic characteristics of included households using χ–square tests. Principal Components Analysis (PCA) has become the norm for classification of households into wealth categories in low resource settings [19]. Households were classified into wealth quintiles using the PCA technique for each survey round to ensure that the same relative level of economic well–being was assessed across study phases. Wealth quintile 1 (Q1) represents the poorest 20% of households in the survey sample and quintile 5 (Q5) represents the least poor. Multivariable logistic regression analysis was used to address the evaluation question of whether differences in the proportion of respondents differed significantly across socioeconomic strata over time for indicators of disease burden, knowledge of zinc/ ORS, careseeking, and treatment practices for diarrhea. To model the prevalence of the various outcome variables and any changes from baseline to endline surveys a model with an interaction term with time for the variable representing dimension of equity was used: *Probability of outcome f or subject i in village j (yij) = b0 + b1(Wealth group) + b2(Time) + B3(wealth group × time) + bi(other explanatory variables) + u_j_* where u_j_ is the random effects due to the village level clustering. Similar models were created for the other dimensions of equity – ethnicity, sex and mothers’ education. Adjusted means for the outcomes for category of the variable of interest holding the other variables in the models at their mean values are presented. Confidence intervals at 95% are presented.

Concentration curves and indices were used to examine socioeconomic inequalities in the distribution of diarrhea prevalence, careseeking, treatment and cost. Concentration curve plots the cumulative share of the health care utilization (ie, use of zinc) against the cumulative share of households in the population ranked from poorest to richest using asset scores [17,18]. If the concentration curve lies above the 45–degree line (line of equality), then the health care utilization is concentrated among the poor and if the concentration curve falls below the line of equality, then health care utilization is concentrated among the rich [18]. If the concentration curve falls along the line of equality, then health care utilization is equally distributed across groups. Concentration indices (CIs) are obtained from the associated concentration curves as twice the area between a concentration curve and the line of equality. The Relative Concentration Index (RCI) measures the extent to which health care utilization is concentrated among particular social groups. It takes on values between −1, when the population’s health care utilization is concentrated among the poor, and +1, when the utilization is concentrated among the rich. A positive index signifies that the distribution of utilization is higher among the richer groups while a negative index indicates utilization is higher among the poor.

Costs were analysed using linear models without any transformations, even though the data showed skewness to the right and “lumpiness” at 0. Using linear models for cost data are considered acceptable when the primary purpose of the analysis is the estimation of average costs [20]. When analyzing costs associated with diarrhea, we have an issue of selection bias – only subjects with diarrhea can incur costs and costs are zero for those who do not suffer from diarrhea. Use of linear models to only those who suffer diarrhea raises the possibility of sample selection bias. The probability of suffering from diarrhea can be influenced by wealth and demographic characteristics which may also influence costs incurred. It is possible that the wealthier groups suffer fewer episodes of illness and may be under sampled if the sample were restricted to only those who suffered from diarrhea. A Heckman sample selection model helps to use the entire sample by first modeling the probability of diarrhea and then using the probability as a predictor of costs. The selection model is based on the notion that some of the independent variables that predict the probability of suffering from diarrhea are different from the independent factors that are associated with costs. The two–step estimation approach is used when the outcome of interest is an observed continuous variable (cost). To assess differences in mean out of pocket payments for treatment and careseeking across socioeconomic strata prior to and after program initiation, we theorized two interdependent models – first, a probit selection model where *Probability of diarrhea (P_d_) = b_0_ + b_Wealth_ + b_Moher’s education_ + b_number of children in the family_ + b_type of family_ + error* and the second is a regression model *Out of pocket cost(C) = b_Wealth_ + b_program intervention_ + b_Wealth_ × b_program intervention_ if P_d_>0* and *Out of pocket cost(C) = 0 if P_d_ = 0.*

We generated mean costs incurred for the different wealth categories and estimated the difference in the costs saved by the intervention. Clustering at the level of the village (Primary Sampling Unit) was accounted for by the use of robust variance estimators based on a first–order Taylor series linear approximation.

### Ethical approval and study status

Ethical approval was obtained from the Johns Hopkins University Bloomberg School of Public Health Institutional Review Board (IRB) and Society for Applied Studies Ethics Research Committee (ERC).

## RESULTS

### Sample characteristics

**Figure 2:**
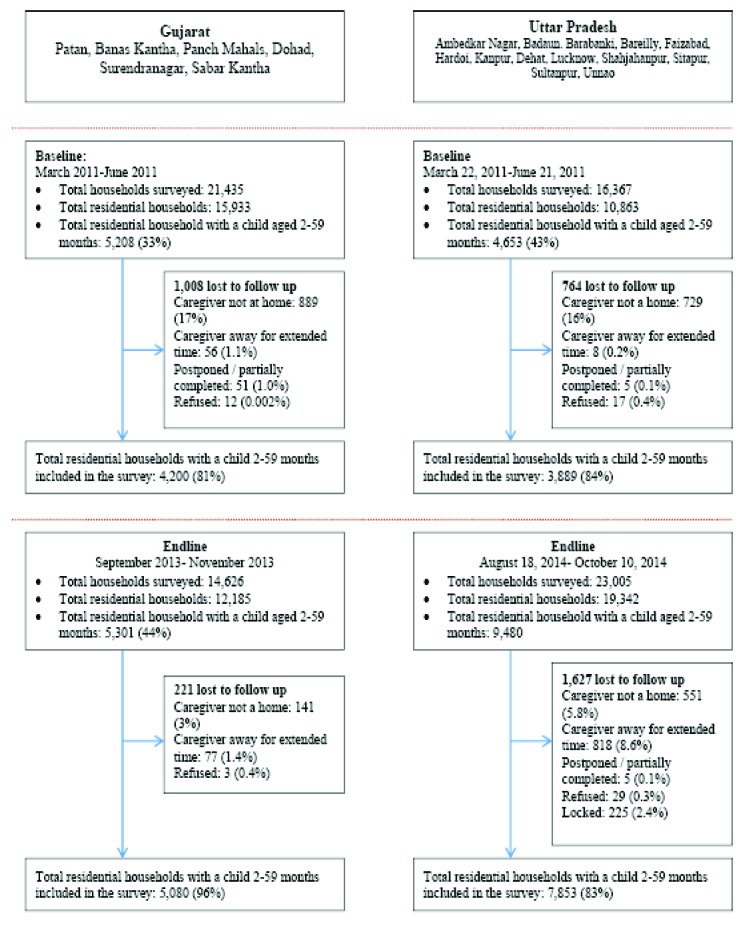
Sampling for baseline and endline household surveys in Gujarat and Uttar Pradesh (UP), India.

Baseline and endline household survey data were collected from 4200 and 5080 caregivers in Gujarat and from 3889 and 7853 caregivers in UP, respectively (**Figure 2**). In both states, household, caregiver, and child characteristics were similar for most parameters assessed (**Table 1**). In Gujarat, significant differences from baseline to endline were observed in the number of nuclear families (37% vs 32%; *P* < 0.001), the proportion of mothers with greater than 1 year of schooling (51% vs 59%; *P* = 0.01), mean number of children under 5 living in the house (1.46 vs 1.40; *P* < 0.001), and the proportion of households below the poverty line (40% vs 48%; *P* < 0.001). In UP, significant differences in the proportion of mothers with greater than 1 year of schooling (38% vs 51%; *P* < 0.001), mean number of children under 5 living in the house (1.44 vs 1.41; *P* = 0.05), the proportion of children breastfed within the previous 24 hours (67% vs 64%; *P* < 0.001), and the proportion of households below the poverty line (28% vs 22%; *P* < 0.001) from baseline to endline.

**Table 1:** Demographic and socioeconomic characteristics of included households

Characteristics	Gujarat (95% confidence interval)			Uttar Pradesh (95% confidence interval)		
**Baseline**	**Endline**	**Endline–Baseline *P* value**	**Baseline**	**Endline**	**Endline–Baseline *P* value**
March–May 2011 (n = 4200)	October–December 2013 (n = 5080)		March–June 2011 (n = 3889)	August–October 2014 (n = 7853)	
Number of nuclear families	37% (35 to 40%)	32% (30 to 34%)	<0.001	55% (52 to 58%)	52% (50 to 54%)	0.16
No. mothers with ≥1 year of schooling	51% (47 to 56%)	59% (56 to 62%)	0.01	38% (34 to 42%)	51% (48 to 53%)	<0.001
No. children <5 living in this house	1.46 (1.43 to 1.49)	1.40 (1.37 to 1.43)	0.00	1.44 (1.41 to 1.47)	1.41 (1.39 to 1.43)	0.05
Mean number boys	0.76 (0.74 to 0.79)	0.74 (0.72 to 0.77)	0.31	0.74 (73 to 77%)	0.73 (72 to 75%)	0.30
Mean number girls	0.70 (0.68 to 0.73)	0.66 (0.63 to .68)	0.01	0.69 (67 to 72%)	0.68 (66 to 69%)	0.30
Age of the index child (mean in months)	24.3 (23.7 to 24.9)	24.0 (23.4 to 24.5)	0.43	24.7 (24.1 to 25.4)	25.3 (24.9 to 25.6)	0.13
Sex of index child: female	46% (44 to 47%)	44% (42 to 45%)	0.10	47% (46 to 49%)	46% (45 to 47%)	0.25
Proportion of children breastfed in the previous 24 h	58% (56 to 60%)	60% (58 to 62%)	0.21	67% (65 to 69%)	64% (63 to 65%)	<0.001
Below poverty line	40% (37 to 44%)	48% (45 to 51%)	0.00	28% (25 to 31%)	22% (21 to 24%)	<0.001
Religion:			0.17			0.54
-Hindu	95% (92 to 98%)	96% (94 to 97%)		88% (83 to 92%)	86% (84 to 88%)	
-Muslim	4% (2 to 9%)	4% (2 to 6%)		12% (8 to 17%)	14% (12 to 16%)	
-Other	0% (0 to 1%)	0% (0 to 1%)		0% (0 to 0%)	0% (0 to 0%)	
Ethnic group:			0.14			0.86
-Scheduled caste	12% (9 to 16%)	17% (14 to 20%)		35% (31 to 40%)	37% (34 to 40%)	
-Scheduled tribe	31% (24 to 38%)	25% (21 to 31%)		0% (0 to 1%)	0% (0 to 0%)	
-Other backward caste	41% (35 to 48%)	44% (40 to 49%)		48% (43 to 53%)	47% (44 to 50%)	
-Other	16% (12 to 21%)	13% (11 to 16%)		17% (13 to 21%)	16% (14 to 18%	

### Caretaker awareness of zinc and ORS

**Figure 3:**
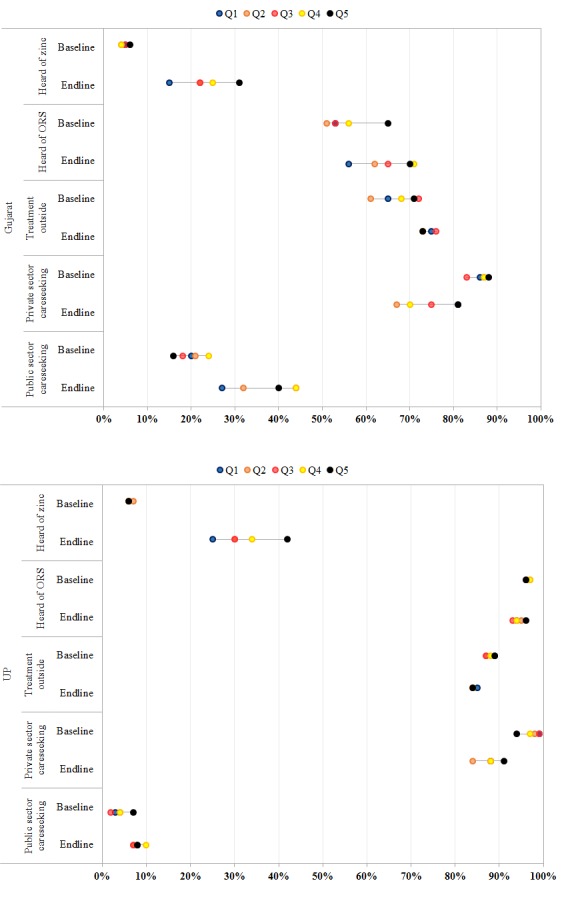
Awareness and careseeking for diarrhea in children 2–59 months in Gujarat and Uttar Pradesh (UP), India.

Caretaker awareness is often viewed as a predictor for treatment seeking behavior. Variations in awareness of zinc and ORS as well as careseeking overall and by sector for diarrhea treatment are presented in **Figure 3** and Table S1 in **Online Supplementary Document(Online Supplementary Document)**. Findings suggest that in contrast to ORS, where nearly all caregivers in UP and half of caregivers in Gujarat reported some baseline knowledge of ORS, baseline awareness of zinc for diarrhea was lower at 6% in UP and 5% in Gujarat. Across socioeconomic strata, a significant 10%–24% increase in zinc awareness was observed over time in Gujarat and 20–36% increase in UP. In both sites, while improvements in zinc awareness were similar by gender and ethnicity, increases were greatest amongst caregivers with ≥1 year of schooling (Gujarat: 22% *P* < 0.001; UP: 30% *P* < 0.001) and in the Q5 socioeconomic strata (Gujarat: 24% *P* < 0.001; UP: 36% *P* < 0.001). In both sites, concentration indices show a pro–rich bias.

### Diarrhea careseeking

Careseeking outside the home for children under 5 with diarrhea did not increase significantly over time in either site across socioeconomic strata or by maternal education (**Figure 3**, Table S3 in **Online Supplementary Document(Online Supplementary Document)**). In Gujarat, careseeking outside the home increased significantly for male children (8%, *P* < 0.001) and members of other backward castes (12%, *P* < 0.001). Across public and private sectors, the majority of careseeking among children with diarrhea occurred in the private sector. However, declines in private sector careseeking were observed over time from 86% to 75% in Gujarat (*P* < 0.00) and from 98% to 87% in UP (*P* < 0.00). Declines in private sector utilization were offset by increases in facility and community–based careseeking in both states. In Gujarat, utilization of public sector providers increased significantly irrespective of maternal education, for both male (19%, *P* < 0.001) and female (16%, *P* < 0.001) children, members of the scheduled (23%, *P* < 0.01) and other backward castes (23%, *P* < 0.01), and amongst members of the Q3 (25%, *P* < 0.001), Q4 (20%, *P* < 0.01), and Q5 (24%, *P* < 0.001) socioeconomic strata (**Figure 3**, Table S2 in **Online Supplementary Document(Online Supplementary Document)**). Among public sector providers, increases in careseeking were driven by increased utilization of community based AWWs and ASHAs. In UP, increases in public sector utilization were significant for male children (5%; *P* = 0.035) and members of the scheduled caste (8%; *P* = 0.004). Increases were similarly driven by a modest increase of 1–4% across all socioeconomic strata in community based careseeking from ASHAs and AWWs.

### Treatment among children with diarrhea

In Gujarat, zinc, ORS and zinc+ORS use increased significantly across all subgroups including socioeconomic strata, gender, ethnicity and maternal education (**Table 2**, Figures S1 and S2, and Table S3 in **Online Supplementary Document(Online Supplementary Document)**). However, the magnitude of increase was lowest among individuals in the Q1 and Q2 socioeconomic strata, members of the disadvantaged scheduled castes and tribe, and among children whose mothers had <1 year of schooling. This trend was similar across both private and public sectors sources for careseeking (**Table 2**). The magnitude of increase from baseline to endline in the number of caregivers receiving ORS from the public sector (23%, from 8% to 31%) was nearly twice that observed in the private sector (12%, from 18% to 30%). Receipt of zinc was similar across sectors, increasing significantly from 1% to 18% (*P* < 0.001) in the public sector and 3% to 18% in the private sector (*P* < 0.001). Increases in the use of home fluids, antibiotics, and antidiarrheals, coupled with declines in use of unknown tablets, powders, and syrups were observed across all sub–groups from baseline to endline.

**Table 2:** Treatment received among children 2–59 month with diarrhea in the preceding 2 weeks by sector

	Gujarat (95% confidence interval)*				Uttar Pradesh (95% confidence interval)†					
**Baseline (n = 594)**	**Endline (n = 553)**		**Endline–Baseline Difference**	**Baseline (n = 572)**		**Endline (n = 854)**			**Endline–Baseline difference**
**Receipt of zinc – public sector**										
Socioeconomic status:										
-Quintile 1	0%	10%	10% (–2 to 21%)		0%	2%				2% (–1 to 4%)
-Quintile 2	1%	9%	8% (2% to 15%)‡		0%	1%				1% (–1 to 3%)
-Quintile 3	2%	18%	16% (8 to 25%)‡		1%	1%				–0% (–3 to 3%)
-Quintile 4	1%	29%	28% (17 to 38%)‡		0%	3%				3% (–0 to 7%)**
-Quintile 5	3%	28%	25% (12 to 38%)‡		0%	3%				3% (–1 to 7%)
Concentration index	0.12 (–0.11 to 0.36), 0.17 (0.00 to 0.34)				0.26 (–0.50 to 1.02), 0.23 (–0.02– to 0.49)					
Gender:										
-Male	1%	21%	15% (9 to 21%)‡		0%	2%			2% (0 to 4%)*	
-Female	0%	33%	20% (14 to 26%)‡		0%	1%			1% (–1 to 3%)	
Ethnicity:										
-Scheduled caste	2%	12%	14% (7 to 21%)‡		0%	1%			1% (–0 to 3%)†	
-Scheduled tribe	3%	16%	12% (3 to 21%)‡		–	–			–	
-Other backward caste	1%	22%	21% (14 to 29%)‡		1%	2%			1% (–1 to 4%)	
-Other	0%	25%	24% (10 to 38%)‡		0%	2%			2% (–1 to 6%)	
Maternal education:										
-Mothers with <1 year of schooling	3%	20%	17% (10 to 24%)‡		0%	2%			2% (0 to 4%)*	
-Mothers with ≥1 year of schooling	1%	17%	18% (12 to 24%)‡		0%	1%			1% (–1 to 3%)	
**Receipt of zinc – private sector**										
Socioeconomic status:										
-Quintile 1	2%	11%	9% (2 to 17%)‡		3%	6%			4% (–1 to 8%)	
-Quintile 2	3%	9%	6% (–2% to 14%)‡		3%	9%			6% (–0 to 12%)§	
-Quintile 3	2%	18%	16% (7% to 24%)‡		5%	7%			3% (–5 to 10%)	
-Quintile 4	3%	22%	19% (9 to 29%)‡		7%	8%			1% (–7 to 9%)	
-Quintile 5	4%	33%	28% (15 to 41%)‡		1%	8%			6% (–1 to 14%)	
Concentration index	0.11 (–0.21 to 0.44), 0.23 (0.10 to 0.36)				0.04 (–0.18 to 0.25), 0.03 (–0.10 to 0.17)					
Gender:										
-Male	4%	20%	16% (9%– to 23%)‡		4%	8%				4% (–1 to 8%)§
-Female	1%	16%	15% (9 to 20%)‡		3%	7%				4% (–1 to 8%)§
Ethnicity:										
-Scheduled caste	1%	21%	20% (9 to 31%)‡		4%	5%				2% (–3 to 6%)
-Scheduled tribe	2%	12%	10% (4 to 16%)‡		–	–				–
-Other backward caste	4%	19%	15% (7 to 2%)‡		5%	9%				4% (–1 to 9%)
-Other	2%	27%	25% (9 to 41%)‡		3%	9%				6% (–2 to 13%)
Maternal education:										
-Mothers with <1 year of schooling	6%	20%	15% (9 to 21%)‡		2%	7%				5% (1 to 8%)‡
-Mothers with ≥1 year of schooling	0%	16%	16% (10 to 21%)‡		6%	8%				2% (–3 to 8%)
**Receipt of ORS – public sector:**										
Socioeconomic status:										
-Quintile 1	5%	22%	18% (5 to 31%)‡		3%	5%				2% (–3 to 6%)
-Quintile 2	9%	24%	15% (3%–27%)‡		1%	4%				3% (–1 to 7%)
-Quintile 3	8%	33%	25% (13 to 37%)*		0%	3%				3% (0 to 6%)‡
-Quintile 4	9%	38%	29% (16 to 42%)*		2%	6%				4% (–2%–10%)
-Quintile 5	10%	37%	27% (11%–42%)*		–1%	4%				5% (–0 to 9%)§
Concentration index	0.19 (0.03 to 0.36), 0.04 (–0.06 to 0.15)				–0.17 (–0.58 to 0.24), 0.15 (–0.06 to 0.36)					
Gender:										
-Male	6%	29%	23% (16 to 31%)		1%	4%				3% (1 to 6%)‡
-Female	10%	33%	23% (14 to 32%)		2%	4%				3% (–1 to 6%)
Ethnicity:										
-Scheduled caste	5%	29%	23% (11 to 35%)‡		1%	5%				4% (0 to 7%)‡
-Scheduled tribe	8%	26%	18% (7 to 29%)‡		–	–				–
-Other backward caste	7%	34%	27% (18 to 37%)‡		1%	3%				2% (–1 to 5%)
-Other	13%	36%	23% (6 to 40%)‡		2%	7%				4% (–1 to 10%)
Maternal education:										
-Mothers with <1 year of schooling	7%	30%	23% (15 to 32%)‡		1%	4%				3% (–0 to 5%)§
-Mothers with ≥1 year of schooling	9%	32%	23% (15 to 31%)‡		1%	4%				3% (0 to 6%)‡
**Receipt of ORS – private sector:**										
Socioeconomic status:										
-Quintile 1	22%	21%	–1% (–11 to 10%)		26%	23%		–3% (–17 to 10%)		
-Quintile 2	17%	19%	2% (–10 to 14%)		22%	25%		3% (–8 to 13%)		
-Quintile 3	19%	29%	9% (–5 to 23%)		26%	15%		–11% (–23 to –0%)‡		
-Quintile 4	16%	39%	23% (9% to 37%)‡		22%	25%		3% (–11 to 16%)		
-Quintile 5	15%	47%	32% (15 to 48%)‡		29%	29%		1% (–14 to 15%)		
Concentration index	0.04 (–0.07 to 0.15), 0.16 (0.07 to 0.24)				0.00 (–0.08 to 0.08), 0.12 (0.05 to 0.20)					
Gender:										
-Male	16%	32%	16% (7 to 26%)‡		23%	26%		3% (–5 to 11%)		
-Female	20%	28%	7% (–2 to 17%)		27%	19%		–8% (–16 to 1%)§		
Ethnicity:										
-Scheduled caste	5%	29%	23% (11 to 35%)‡		23%	23%		–0% (–11 to 10%)		
-Scheduled tribe	8%	26%	18% (7 to 29%)‡		–	–		–		
-Other backward caste	7%	34%	27% (18 to 37%)‡		26%	21%		–5% (–14 to 3%)		
-Other	13%	36%	23% (6 to 40%)‡		23%	29%		5% (–7 to 18%)		
Maternal education:										
-Mothers with <1 year of schooling	16%	28%	12% (4 to 20%)‡		22%	18%		–4% (–12 to 5%)		
-Mothers with ≥1 year of schooling	20%	31%	12% (2 to 21%)‡		28%	29%		1% (–8 to 9%)		

*Adjusted for type of family, maternal education, number of children, below poverty, and ethnicity.

†Adjusted for type of family, maternal education, number of children, gender, age in mothers, breastfeeding status, below poverty, and ethnicity.

‡*P* < 0.05.

§*P* = 0.10.

In UP, significant increases from baseline to endline across socioeconomic strata in zinc and ORS use were not observed, while the use of antidiarrheals increased significantly from 48% in the Q1 (*P* < 0.001) to 34% in the Q5 (*P* < 0.001) (Table S3 in **Online Supplementary Document(Online Supplementary Document)**). When stratified by sector, zinc receipt increased in the private sector (from 4% to 8%; *P* = 0.03) and ORS increased in the public sector (from 1% to 4%; *P* = 0.001). Increases in antidiarrheals was highest amongst female children (43% vs 38% for males), members of scheduled and other castes, and for children of mother’s with <1 year of schooling (43% vs 36%). The use of antibiotics was reported by nearly one–third of caregivers at baseline and endline and did not differ significantly across socioeconomic strata over time. Over the same time period, utilization of other treatment, including unknown powders, pills and syrups, declined significantly over time across all income strata and in particular amongst the least poor individuals.

### Treatment among children with severe dehydration and diarrhea

**Figure 4:**
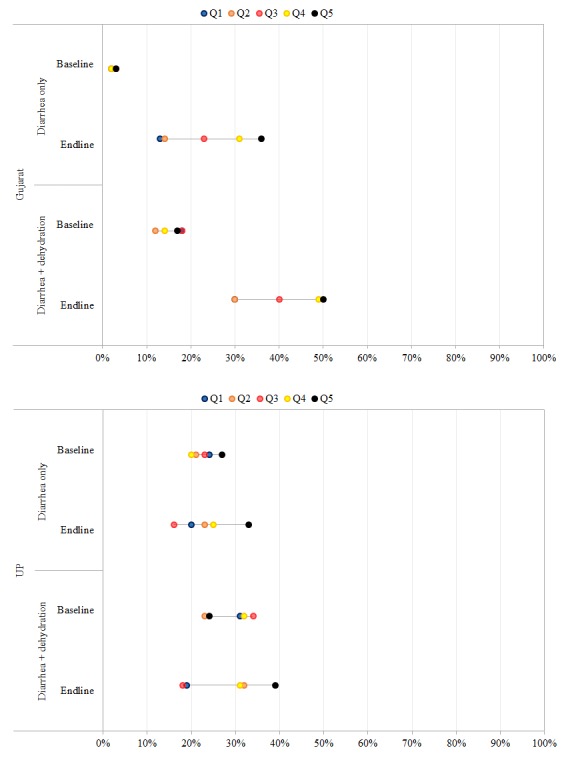
ORS use amongst children with diarrhea vs those with dehydration and diarrhea in the preceding 2 weeks at baseline and endline in Gujarat and Uttar Pradesh (UP), India.

To measure vertical equity and ensure that program activities did not have an adverse effect on the treatment of children with dehydration in addition to diarrhea, we sought to assess changes in ORS use over time in both sites for children with diarrhea alone vs those with dehydration in addition to diarrhea in the preceding 2 weeks (**Figure 4**). In Gujarat, ORS coverage rates prior to DAZT introduction, were similar for children with diarrhea (16%) and those with dehydration + diarrhea (17%). However, at endline, ORS use increased significantly for children with diarrhea (23%, *P* < 0.001) and those with dehydration + diarrhea (33%, *P* < 0.001). Across socioeconomic strata, the magnitude of increase was highest amongst children in the Q5 income strata in both groups. In UP, ORS use rates for both children with diarrhea and those with dehydration + diarrhea did not change significantly from baseline to endline across socioeconomic strata.

### Beneficiary costs per patient

**Table 3** presents data on the mean out of pocket payments for diarrheal episodes in the preceding 2 weeks in 2014 US dollars by subgroup. In Gujarat, the mean cost among children with diarrhea who sought care declined significantly from US$ 5.97 to US$ 4.01 (*P* < 0.001) from baseline to endline and significantly amongst the least poor (–US$ 4.24, *P* < 0.003). Out of pocket spending was nearly US$ 1 higher for male children at baseline and US$ 0.59 at endline. Over time, costs to users declined by a mean of US$ 2 from baseline to endline, largely as a result of significant reductions in reported wages lost (–US$ 0.79; *P* < 0.003), and transportation costs (–US$ 0.44; *P* < 0.00). The magnitude of declines were greatest amongst the least poor for both wages lost (–US$ 1.57, *P* = 0.005), and transportation (US$ 0.86, *P* < 0.003) from baseline to endline. Concentration indices similarly suggest a significant pro–rich bias with the greatest reductions in cost occurring amongst the least poor, however, the magnitude of difference across socioeconomic strata decreased at endline. In UP, mean costs for diarrhea treatment among those who sought care did not change significantly over time across socioeconomic strata.

**Table 3:** Total costs in USD for diarrhea treatment among those who seek care in the preceding 2 weeks

	Gujarat (95% confidence interval)			Uttar Pradesh (95% confidence interval)				
**Baseline (n = 398)**	**Endline (n = 412)**	**Endline–Baseline Difference**	**Baseline (n = 572)**		**Endline (n = 854)**		**Endline–Baseline difference**
Total cost	5.97	4.01	–1.96 (–3.29 to –0.63)*	4.61		4.99		0.38 (–0.41–1.17)
Socioeconomic status:								
-Quintile 1	3.91	3.03	–0.88 (–3.28 to 1.52)	4.83		5.16		0.33 (–1.84 to 2.49)
-Quintile 2	4.55	3.37	–1.17 (–3.61 to 1.26)	3.44		5.11		1.67 (–0.46 to 3.80)
-Quintile 3	7.29	4.05	–3.33 (–5.56 to –0.92)*	5.89		4.3		–1.58 (–3.75 to 0.58)
-Quintile 4	4.25	4.26	0.02 (–2.34 to 2.38)	6.37		6.27		–0.11 (–2.38 to 2.17)
-Quintile 5	8.18	3.94	–4.24 (–7.02 to –1.46)*	4.98		5.77		0.79 (–1.79 to 3.38)
Concentration index	0.10 (–0.00 to 0.19), 0.04 (–0.03 to 0.10)			0.05 (–0.03 to 0.12), 0.07 (0.01 to 0.12)				
Gender:								
-Male	6.46	4.44	–2.01 (–3.54 to –0.49)*	4.38	5.53		1.14 (–0.23 to 2.52)	
-Female	5.42	3.85	–1.57 (–3.17 to 0.02)*	4.37	3.48		–0.89 (–2.37 to 0.59)	
Ethnicity:								
-Scheduled caste	4.73	4.49	–0.24 (–2.85 to 2.37)	4.86	4.36		–0.50 (–2.19 to 1.19)	
-Scheduled tribe	5.83	4.48	–1.36 (–3.32 to 0.60)					
-Other backward caste	5.99	3.12	–2.87 (–4.62 to –1.12)*	4.25	5.19		0.94 (–0.50 to 2.37)	
-Other	6.05	4.29	–1.76 (–5.07 to 1.55)	5.29	4.76		–0.54 (–3.07 to 2.00)	
Maternal education:								
-Mothers with <1 years of schooling	7.40	4.52	–2.88 (–4.49 to –1.27)*	3.80	4.87		1.07 (–0.29 to 2.43)	
-Mothers with ≥1 years of schooling	5.49	4.65	–0.84 (–2.36 to 0.69)	5.87	5.00		–0.87 (–2.38 to 0.64)	

*P < 0.05.

### Diarrhea prevalence

**Figure 5:**
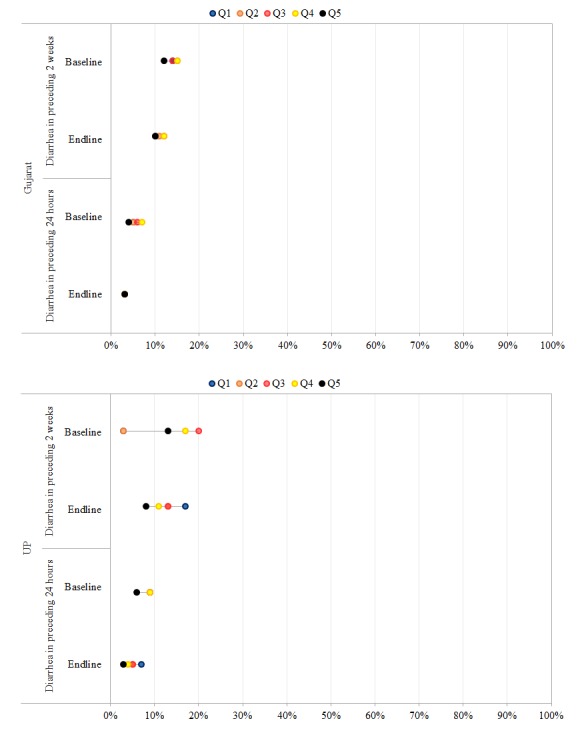
Diarrhea prevalence and incidence in Gujarat and Uttar Pradesh (UP), India.

**Figure 5** presents data on the differences in the period prevalence of diarrhea in the preceding 24 hours and in the preceding 2 weeks. In Gujarat, the proportion of children with diarrhea in the preceding 24 hours declined significantly across all sub–groups. The magnitude of decline in prevalence in the last 24 hours and 2 weeks was greatest for children in the Q1 and Q2 socioeconomic strata, among the least disadvantaged castes, and with mothers with <1 year of schooling. Despite declines in diarrhea prevalence, concentration indices suggest a pro–poor bias, indicating higher disease burden amongst the poorest at endline (–0.13,95% confidence interval (CI) –0.17 to –0.08) vs baseline (–0.04, 95% CI –0.08 to 0.01). In UP, significant declines were observed in the proportion of children with diarrhea in the preceding 2 weeks and 24 hours across all sub–groups, with the exception of the poorest. Concentration indices for diarrhea prevalence in the preceding 2 weeks and 24 hours mirror those for Gujarat and suggest a significant pro–rich bias over time indicating higher disease burden amongst the poorest at endline (–0.01, 95% CI –0.06 to 0.04) vs baseline (0.16, 96% CI 0.12 to 0.20).

## DISCUSSION

Overall study findings suggest that DAZT programmatic activities corresponded to a significant increase in caregiver awareness of zinc in Gujarat (18%, *P* < 0.001) and UP (26%, *P* < 0.001) across all dimensions of equity considered, including socioeconomic strata, gender, ethnicity and caregiver education. Careseeking for diarrhea treatment outside the home did not increase significantly across socioeconomic strata in either state, however, a significant increase was observed in Gujarat for male children, members of other backward castes, and children whose mothers had ≥1 year of schooling. While the private sector constituted over 80% of careseeking in both states, slight declines (~ 10%) were observed over time coinciding with an increase in public sector utilization (Gujarat: 17% increase from 20%–37%; UP: 4% increase from 4%–8%). Increases in use of zinc, ORS, and zinc+ORS, were only observed in Gujarat despite a longer period of implementation in UP. The magnitude of increase in Gujarat was lowest amongst children in the Q1 and Q2 income strata, female children, members of the scheduled tribe, and children of mothers with <1 year of schooling. Further increases in antibiotic (17–23%) and antidiarrheal (16–24%) use occurred for 4 of 5 socioeconomic strata along with significant increases in the median number of treatments taken. Overall, modest gains in awareness (both sites), and product use (Gujarat only) were disproportionately observed amongst the least poor; suggesting a pro–rich bias and trend towards ‘inverse equity’ reported elsewhere [21].

Study findings fall well beneath the effectiveness of smaller scale efforts to increase zinc and ORS use in Haryana which corresponded to zinc and ORS use in 60% and 59% of diarrhea cases in the preceding 4 weeks after 9 months of implementation [22]. However, findings are similar to those reported from efforts to deliver zinc at scale in Bangladesh through the Scaling Up of Zinc for Young Children (SUZY) Project [23]. SUZY activities in Bangladesh were implemented over a 3–year period (2003–2006) through public and private delivery channels, resulting in increased awareness and zinc use in four populations: city slum, city non–slum, municipal, and rural [23]. Across socioeconomic strata, SUZY activities were similarly associated with a pro–rich bias, reaching a peak of just under 30% among the least poor vs ~ 7% amongst the poorest at 7–10 months of implementation [23,24]. Amongst the least poor in Gujarat, zinc utilization under DAZT reached a peak of 36% at endline (reflecting a 33% increase from baseline), while ORS use rose from 17% to 50% from baseline to endline. Use rates among the least poor mirror those reported in Bangladesh, suggesting an increase from 2% to 13% from baseline to endline for zinc and 18% to 30% for ORS.

Despite the similarities in the effectiveness of efforts to scale zinc in Bangladesh and Gujarat, programmatic activities in UP did not yield significant improvements in zinc and ORS use. DAZT study findings may be attributed either to a negative confounding due to a worsening of contextual factors or alternatively effect modification [25]. We sought to measure potential confounders and effect modifiers through the household survey as well as efforts during analysis to adjust for observed differences in population health status, characteristics, and practices. However, implementation–related effect modifiers were much more challenging to measure and understand, including differences across and within states in the health service characteristics related to supply, availability of human resources, and supervision, as well as the presence of other programs.

In UP and Gujarat, the overwhelming majority of treatment–seeking occurred in the private sector. While private sector engagement has been reported elsewhere in India with much success [22], the scale of prior implementation efforts has been significantly less than that achieved under DAZT. While DAZT programmatic efforts corresponded to the training of thousands of health workers, private sector engagement was not precipitated with a formal census and thus the true denominator from which DAZT–engaged providers were identified remains unknown. Without question, engagement with the private sector is not an easy task – the market is expansive and evolving, and the type and quality of providers varied. However, in the absence of data on the proportion of providers identified, trained, followed–up and prescribing zinc, it remains challenging to ascertain the true supply side coverage of the program. Further challenges associated with village level supply monitoring too hampered efforts to fully capture the depth and breadth of programmatic inputs and their link with outcomes reported here.

DAZT implementation targeted rural areas where access to basic health services as well as adequate water and sanitation facilities were more limited [25]. Even within the rural areas, trends in inverse equity – which postulates that zinc and ORS uptake would occur first by the wealthiest, leading to increased inequities, before uptake occurs amongst the poorest – were observed [21]. The focus on rural populations limits the generalizability of study findings to other geographic areas and population groups; potentially masking wider disparities likely to occur in more heterogeneous implementation.

There is precedence for our approach to measuring economic poverty through wealth quintiles [7,13,14,25,26]. However, in the case of principal components analyses, the quality of assets are not considered, the first principal component is assumed to be an adequate indicator of socioeconomic status, and ultimately quintiles are produced with variable arrays of asset ownership [14,19,27]. There are further limitations to multiple approaches that operate under an additive assumption [28]. In an effort to address the limitations of measuring equity singularly through the lens of socioeconomic status, we expanded the dimensions assessed to include education, gender, ethnicity and geography, and sought to identify determinants of inequities in disease burden, careseeking, and practices related to diarrheal diseases. Elsewhere inequities in child health disease burden and management have been reported for gender, ethnicity and education [23,24,29]. Findings from Bangladesh highlighted disparities in the likelihood of receiving zinc based on gender in municipal households (21% for males vs 16% for females, *P* = 0.024) [23,24]. Findings from DAZT program activities in Gujarat suggest that overall receipt of zinc is similar for males and female children, however, in the public sector females have a slightly higher use of zinc (33% for women vs 21% for males) and ORS (33% for women vs 29% for males).

An intersectionality perspective would posit that inequities observed are not the result of a single, distinct factor but rather the outcome of the “intersection of different social locations, power relations, and experiences” [28]. Efforts to explore the intersectional inequalities in immunization coverage in India provided important insights into disparities across gender, caste, and place of residence along with wealth [29]. Our inclusion of these parameters and their further expansion to include ethnicity and education was intended to provide greater insights into the inequalities observed and generate clearer evidence that can be used to meaningfully intervene and improve programmatic effectiveness [29,30].

Beyond efforts to expand the range of evaluation methods employed and consider alternative underpinning theoretical approaches for assessing inequity, it is worth noting that the metrics for evaluating coverage in diarrheal disease programs can be unforgiving on the program being assessed. The nature of household surveys for diarrhea management necessitates a short recall window of 2 weeks to limit caregiver recall biases. This arguably sets a high programmatic bar to achieve effective product availability and use; requiring adequate product supplies, provider knowledge and willingness to prescribe, coupled with caregiver demand, affordability, and use within the narrow widow of time under assessment. The approach adopted by the SUZY program of more frequent cross–sectional surveys may allow for greater insights into ongoing program implementation, particularly when linked to specific activities (eg, mass media campaigns), while providing insights into variations in coverage over time.

## CONCLUSION

This paper aims to contribute to a growing body of evidence on inequities in child health [7,13,31,32] and in diarrhea [23]. As one of few equity analyses conducted of a diarrhea treatment program at scale, findings provide further evidence suggesting that magnitude of effect observed under efficacy and effectiveness trials wanes as programs are scaled. Inverse inequities in the number of treatments as well as uptake of ORS and zinc were observed in Gujarat, along with increased use of antibiotics and antidiarrheals. If national–level reductions in diarrheal disease burden are to be realized in India, improved understanding of how to optimally increase coverage of zinc and ORS and decrease contraindicated treatments is essential, particularly amongst the poorest [13].
