# Supplementary material for: Diarrhea no more: does zinc help the poor? Evidence on the effectiveness of programmatic efforts to reach poorest in delivering zinc and ORS at scale in UP and Gujarat, India
Source: J Glob Health. 2016 Dec 14;6(2):021001. doi: 10.7189/jogh.06.021001 (PMC5234496; doi:10.7189/jogh.06.021001)
Supplement: Online Supplementary Document [file jogh-06-021001-s001.pdf]

Online Supplementary Document

LeFevre et al. Diarrhea no more: does zinc help the poor? Evidence on the effectiveness of programmatic efforts to reach poorest in delivering zinc and ORS at scale in UP and Gujarat, India

J Glob Health 2016;6:021001

Figure S1. Treatment for diarrhea in children under 5 by socioeconomic status and state

Gujarat

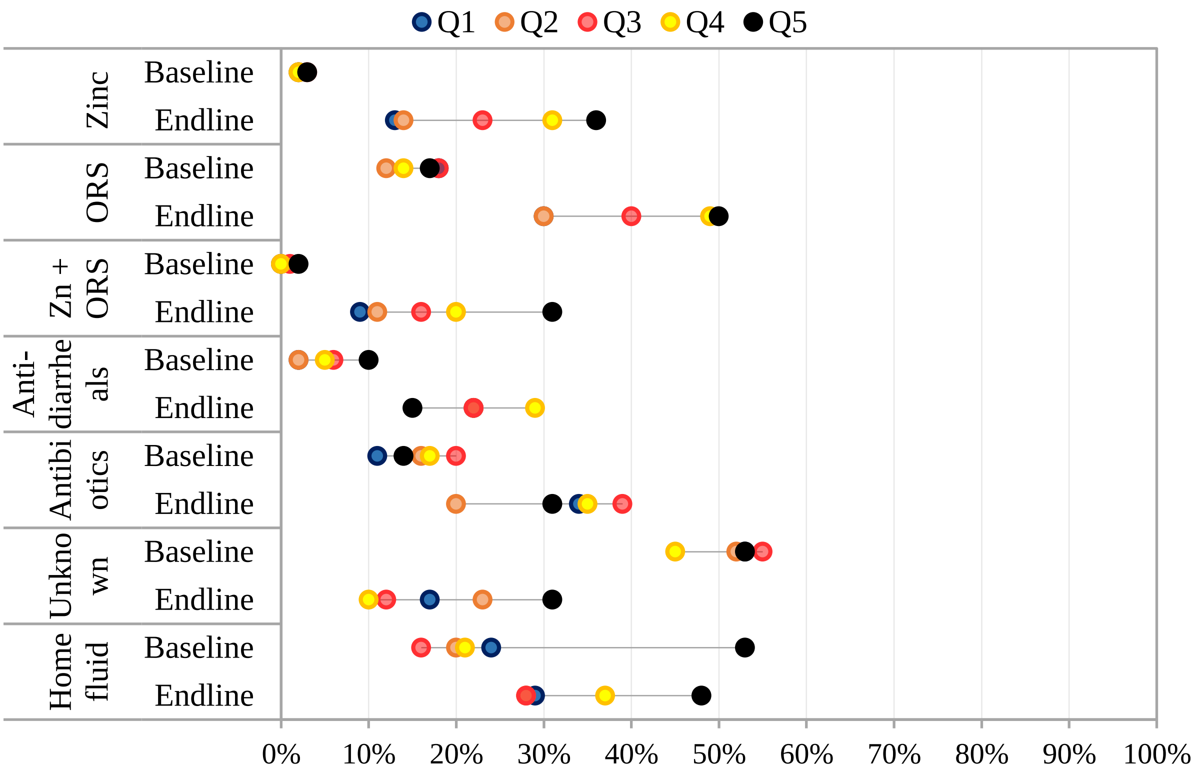

Uttar Pradesh Treatment for diarrhea in children under 5 by socioeconomic status and state

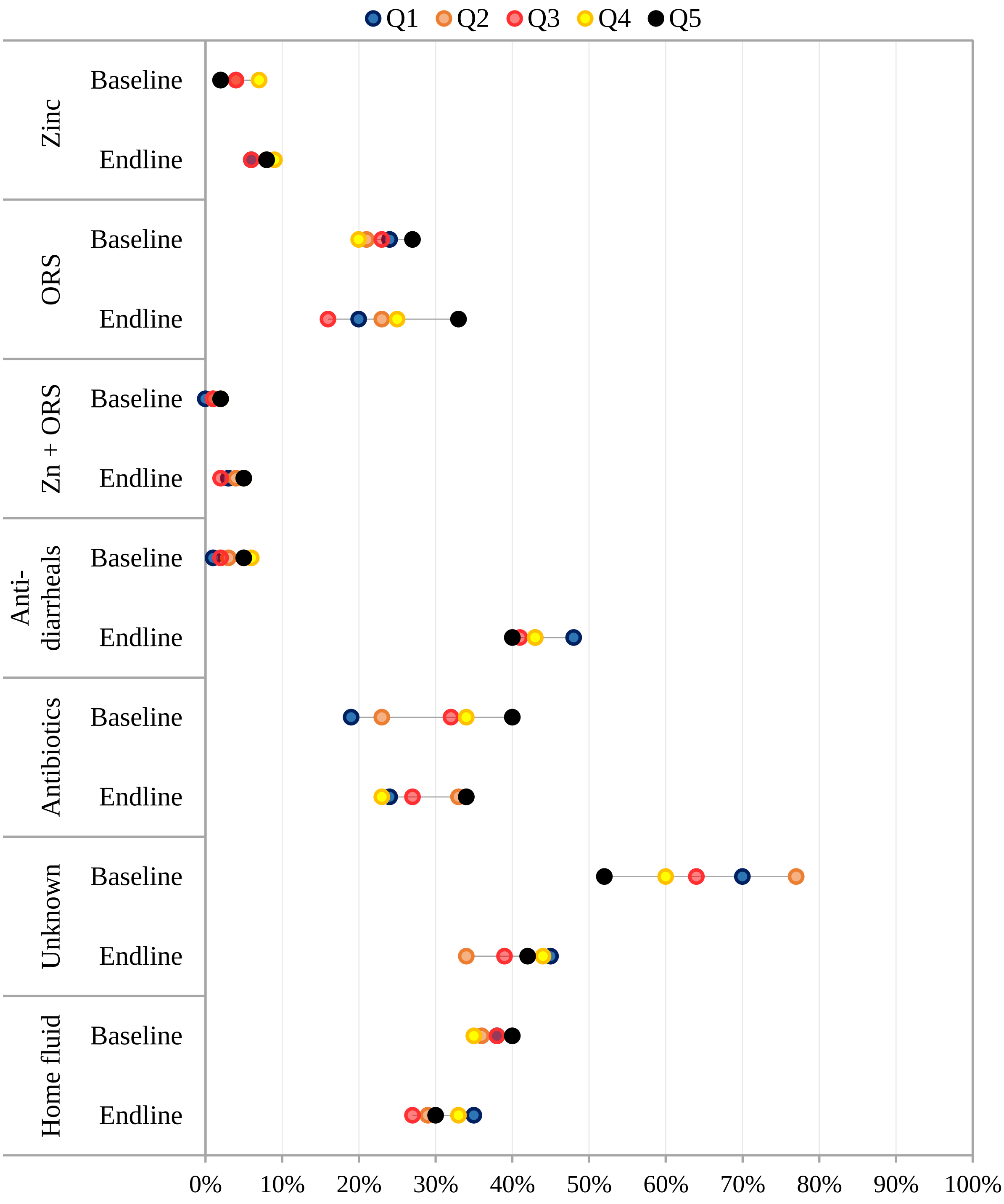
Q1
Q2
Q3
Q4
Q5
Zinc
ORS
Zn + ORS
Anti-diarrheals
Antibiotics
Unknown
Home fluid
Baseline
Endline
0%
10%
20%
30%
40%
50%
60%
70%
80%
90%
100%

Table S1. Awareness of zinc and ORS for the treatment of diarrhea in Gujarat and UP

	Gujarat ***			Uttar Pradesh****		
	**Baseline**	**Endline**	**Endline-Baseline Difference**	**Baseline**	**Endline**	**Endline-Baseline Difference**
	n=4,200	n=5,080		n=3,889	n= 7,853	
**Heard of zinc for diarrhea***						
**Socioeconomic status**						
Q1	5%	15%	10% (6%-13%)*	6%	25%	20% (16%-23%)*
Q2	4%	22%	18% (14%-22%)*	7%	30%	22% (18%-27%)*
Q3	5%	22%	17% (14%-21%)*	6%	30%	24% (20%-28%)*
Q4	4%	25%	21% (16%-25%)*	6%	34%	27% (23%-31%)*
Q5	6%	31%	24% (19%-30%)*	6%	42%	36% (32%-41%)*
Concentration index (95% CI)	0.21 (0.10-0.32)	0.14 (0.09-0.19)		0.01 (-0.07-0.10)	0.02 (0.00-0.04)	
**Gender**						
Male	5%	23%	18% (15%-21%)*	7%	31%	24% (21%-27%)*
Female	4%	22%	18% (15%-21%)*	5%	33%	28% (25%-32%)*
**Ethnicity**						
Scheduled caste	4%	25%	21% (15%-26%)*	7%	31%	25% (21%-28%)
Scheduled tribe	5%	19%	14% (10%-18%)*	[redacted]	[redacted]	
Other backward caste	6%	25%	19% (15%-22%)*	7%	32%	25% (21%-28%)*
Other	3%	23%	20% (15%-26%)*	5%	35%	31% (26%-35%)*
**Maternal education**						
Mothers with <1 year of schooling	5%	17%	13% (10%-16%)*	7%	29%	22% (20%-25%)*
Mothers with ≥1 year of schooling	5%	27%	22% (19%-26%)*	5%	35%	30% (27%-33%)*
**Heard of ORS for diarrhea**						
**Socioeconomic status**						
Q1	53%	56%	3% (-5%-12%)	96%	94%	-2% (-5%-1%)
Q2	51%	62%	11% (5%-18%)*	97%	95%	-2% (-4%--1%)
Q3	53%	65%	12% (6%-19%)*	96%	93%	-3% (-5%- -0%)*
Q4	56%	71%	15% (9%-21%)*	97%	94%	-3% (-5%- -1%)*
Q5	65%	70%	6% (-1%-12%)	96%	96%	-0% (-2%-1%)

Concentration index (95% CI)	0.06 (0.03-0.08)	0.06 (0.04-0.08)		-0.00 (-0.01- -0.00)	-0.00 (-0.01-0.00)	
**Gender**						
Male	56%	66%	10% (5%-15%)*	96%	94%	-2% (-4%- -0%)*
Female	55%	64%	9% (5%-14%)*	97%	95%	-2% (-4%- -0%)*
**Ethnicity**						
Scheduled caste	60%	75%	15% (8%-22%)*	98%	94%	-3% (-5%- -1%)*
Scheduled tribe	55%	61%	6% (-2%-13%)	[redacted]		
Other backward caste	55%	64%	8% (2%-15%)*	95%	94%	-1% (-3%-1%)
Other	51%	65%	15% (7%-23%)*	98%	96%	-1% (-3%-1%)
**Maternal education**						
Mothers with <1 yr of schooling	49%	59%	8% (1%-15%)*	95%	94%	-2% (-3%-0%)
Mothers with ≥1 yr of schooling	61%	69%	9% (5%-14%)*	98%	95%	-2% (-4%- -1%)*

Table S2. Sources of careseeking for children 2-59 months with diarrhea in the preceding 2 weeks

	Gujarat***			Uttar Pradesh****		
	**Baseline**	**Endline**	**Endline-Baseline Difference**	**Baseline**	**Endline**	**Endline-Baseline Difference**
**Treatment outside the home**	**n=594**	**n=553**		**n=652**	**n=1,001**	
**Socioeconomic status**						
Q1	65%	75%	10% (-3%-22%)	89%	83%	-6% (-14%-2%)
Q2	61%	73%	12% (0%-24%)	91%	89%	-2% (-10%-6%)
Q3	72%	76%	4% (-7%-16%)	85%	78%	-7% (-17%-2%)
Q4	68%	73%	6% (-8%-19%)	89%	85%	-5% (-15%-6%)
Q5	71%	73%	2% (-11%-16%)	87%	90%	3% (-7%-14%)
Concentration index (95% CI)	0.02 (-0.01-0.05)	-0.03 (-0.06-0.00)		-0.01 (-0.02-0.01)	0.01 (0.00- 0.02)	
**Gender**						
Male	67%	76%	8% (1%-16%)*	89%	85%	-4% (-9%-1%)
Female	67%	73%	6% (-2%-14%)	87%	84%	-4% (-10%-3%)
**Ethnicity**						
Scheduled caste	77%	77%	-0% (-12%-12%)	89%	84%	-5% (-12%-2%)
Scheduled tribe	67%	73%	6% (-5%-17%)	-	-	-
Other backward caste	63%	74%	12% (3%-21%)*	87%	86%	-1% (-7%-5%)
Other	71%	74%	3% (-12%-19%)	88%	78%	-10% (-20%- -1%)*
**Maternal education**						
Mothers with <1 yr of schooling	68%	73%	5% (-3%-13%)	89%	84%	-5% (-10%-1%)
Mothers with ≥1 yr of schooling	66%	75%	9% (1%-17%)*	87%	85%	-3% (-9%-4%)
**Private sector careseeking**	**n=398**	**n=412**		**n=572**	**n=854**	
**Socioeconomic status**						
Q1	86%	81%	-5% (-18%-9%)	99%	88%	-11% (-18%- -5%)*
Q2	87%	67%	-20% (-33%- -6%)*	98%	84%	-14% (-21%- -6%)*
Q3	83%	75%	-8% (-20% -4%)	99%	88%	-11% (-17%- -4%)*
Q4	87%	70%	-16% (-30%- -3%)*	97%	88%	-10% (-18%- -2%)*
Q5	88%	81%	-7% (-22%-8%)	94%	91%	-3% (-11%-5%)
Concentration index (95% CI)	-0.00 (-0.02-0.02)	0.01 (-0.03-0.05)		-0.00 (-0.01-0.00)	-0.00 (-0.01-0.02)	
**Gender**						
Male	89%	76%	-13% (-21%- -6%)*	98%	89%	-10% (-14%- -5%)*
Female	82%	74%	-9% (-19%-2%)	97%	86%	-11% (-16%- -6%)*

**Ethnicity**						
Scheduled caste	90%	76%	-14% (-27%- -0%)*	98%	86%	-12% (-18%- -6%)*
Scheduled tribe	88%	78%	-10% (-21%-1%)	[redacted]		
Other backward caste	85%	70%	-15% (-26%- -4%)*	98%	88%	-10% (-15%- -5%)*
Other	81%	83%	3% (-13%-18%)	96%	90%	-6% (-14%-2%)
**Maternal education**						
Mothers with <1 yr of schooling	88%	77%	-10% (-19%--2%)	98%	86%	-12% (-17%- -8%)*
Mothers with ≥1 yr of schooling	84%	73%	-12% (-21%- -3%)	97%	89%	-7% (-12%- -3%)*
**Public sector careseeking**	**n=398**	**n=412**		**n=572**	**n=854**	
**Socioeconomic status**						
Q1	20%	27%	8% (-8%-23%)	3%	8%	5% (-1%-11%)**
Q2	21%	32%	10% (-4%-25%)	4%	7%	4% (-2%-10%)
Q3	18%	44%	25% (12%-39%)*	2%	7%	5% (-1%-11%)
Q4	24%	44%	20% (4%-35%)*	4%	10%	6% (-2%-13%)
Q5	16%	40%	24% (8%-41%)*	7%	8%	1% (-10%-11%)
Concentration index (95% CI)	0.03 (-0.10- 0.15)	0.04 (-0.05- 0.13)		0.13 (-0.05-0.31)	0.15 (0.02-0.27)	
**Gender**						
Male	17%	36%	19% (9%-28%)*	4%	9%	5% (0%-9%)*
Female	23%	39%	16% (5%-27%)*	4%	7%	3% (-2%-8%)
**Ethnicity**						
Scheduled caste	14%	37%	23% (9%-38%)*	3%	10%	8% (3%-13%)*
Scheduled tribe	20%	31%	11% (-2%-24%)	[redacted]		
Other backward caste	19%	42%	23% (11%-34%)*	4%	6%	2% (-3%-6%)
Other	29%	37%	8% (-12%-29%)	6%	9%	3% (-5%-12%)
**Maternal education**						
Mothers with <1 yr of schooling	19%	37%	18% (8%-28%)*	4%	8%	4% (-0%-8%)**
Mothers with ≥1 yr of schooling	21%	38%	17% (7%-27%)*	4%	8%	4% (-0%-9%)**

*p<0.05; **p=0.10;

***Adjusted for type of family, maternal education, number of children, below poverty, and ethnicity; ****Adjusted for type of family, maternal education, number of children, gender, age in mothers, breastfeeding status, below poverty, and ethnicity

Table S3. Treatment received among children 2-59 months with diarrhea in the preceding 2 weeks

Number of diarrhea cases in the preceding 2 weeks	Gujarat			Uttar Pradesh		
	Baseline n=594	Endline n=553	Endline-Baseline Difference	Baseline n=652	Endline n=1,001	Endline-Baseline Difference
**Zinc**						
**Socioeconomic status**						
Q1	2%	13%	11% (1%-20%)*	2%	6%	4% (-0%-8%)**
Q2	2%	14%	12% (5%-19%)*	4%	8%	4% (-2%-10%)
Q3	3%	23%	20% (11%-28%)*	4%	6%	2% (-4%-8%)
Q4	2%	31%	29% (19%-39%)*	7%	9%	2% (-5%-9%)
Q5	3%	36%	33% (22%-44%)*	2%	8%	7% (-0%-14%)**
Concentration index (95% CI)	0.12 (-0.16-0.41)	0.16 (0.05- 0.28)		-0.01 (-0.22-0.21)	0.09 (-0.04-0.21)	
**Gender**						
Male	3%	23%	20% (14%-26%)*	4%	8%	4% (0%-8%)*
Female	1%	22%	21% (15%-26%)*	3%	6%	3% (-1%-6%)
**Ethnicity**						
Scheduled caste	0%	22%	22% (13%-32%)*	4%	5%	1% (-3%-5%)
Scheduled tribe	3%	16%	13% (6%-20%)*	-	-	-
Other backward caste	3%	26%	23% (16%-31%)*	4%	8%	4% (-0%-9%)**
Other	1%	28%	28% (14%-42%)*	3%	8%	5% (-1%-11%)**
**Maternal education**						
Mothers with <1 yr of schooling	5%	23%	18% (12%-24%)*	3%	7%	4% (1%-7%)*
Mothers with ≥1 yr of schooling	0%	22%	22% (17%-28%)*	5%	8%	2% (-3%-8%)
**ORS**						
**Socioeconomic status**						
Q1	18%	30%	12% (0%-24%)*	24%	20%	-5% (-16%-7%)
Q2	12%	30%	17% (6%-29%)*	21%	23%	2% (-8%-11%)
Q3	18%	40%	21% (9%-34%)*	23%	16%	-8% (-18%-1%)
Q4	14%	49%	35% (23%-47%)*	20%	25%	5% (-8%-17%)
Q5	17%	50%	33% (20%-46%)*	27%	33%	6% (-8%-21%)
Concentration index (95% CI)	0.05 (-0.04-0.15)	0.04 (-0.03-0.11)		-0.01 (-0.09-0.07)	0.01 (-0.06-0.08)	
**Gender**						
Male	14%	39%	25% (17%-33%)*	21%	24%	3% (-4%-10%)
Female	17%	39%	22% (13%-30%)*	25%	20%	-5% (-12%-2%)
**Ethnicity**						

Scheduled caste	17%	46%	30% (16%-43%)*	22%	23%	1% (-9%-10%)
Scheduled tribe	16%	31%	15% (4%-27%)*	-	-	-
Other backward caste	15%	41%	26% (17%-34%)*	24%	19%	-4% (-12%-4%)
Other	15%	43%	28% (13%-44%)*	21%	28%	7% (-4%-17%)
**Maternal education**						
Mothers with <1 yr of schooling	14%	36%	23% (14%-31%)*	21%	17%	-3% (-11%-4%)
Mothers with ≥1 yr of schooling	18%	41%	24% (16%-31%)*	25%	27%	2% (-6%-10%)
**Zinc and ORS**						
**Socioeconomic status**						
Q1	0%	9%	9% (0%-17%)*	0%	3%	2% (0%-5%)
Q2	0%	11%	11% (5%-17%)*	1%	4%	3% (-1%-7%)
Q3	1%	16%	16% (8%-23%)*	1%	2%	0% (-2%-3%)
Q4	0%	20%	29% (19%-38%)*	2%	5%	4% (-1%-8%)*
Q5	2%	31%	30% (19%-40%)*	2%	5%	3% (-3%-10%)
Concentration index (95% CI)	0.59 (0.06-1.12)	0.29 (0.05-0.31)		0.13 (-0.17-0.42)	0.04 (-0.15-0.23)	
**Gender**						
Male	0%	19%	19% (14%-25%)*	2%	5%	3% (-0%-5%)**
Female	1%	18%	17% (12%-22%)*	0%	2%	2% (-0%-4%)**
**Ethnicity**						
Scheduled caste	-2%	16%	18% (9%-26%)*	1%	2%	1% (-1%-4%)
Scheduled tribe	2%	14%	12% (5%-19%)*	-	-	-
Other backward caste	0%	21%	21% (15%-28%)*	1%	4%	3% (-1%-6%)
Other	-1%	25%	25% (13%-38%)*	2%	5%	3% (-3%-8%)
**Maternal education**						
Mothers with <1 yr of schooling	2%	18%	16% (10%-22%)*	1%	4%	3% (1%-5%)*
Mothers with ≥1 yr of schooling	-1%	19%	20% (15%-25%)*	2%	3%	2% (-1%-5%)
**Antidiarrheals**						
**Socioeconomic status**						
Q1	2%	22%	19% (10%-28%)*	1%	48%	47% (38%-56%)*
Q2	2%	22%	19% (11%-27%)*	3%	43%	40% (30%-51%)*
Q3	6%	22%	16% (8%-25%)*	2%	41%	39% (30%-48%)*
Q4	5%	29%	23% (14%-33%)*	6%	43%	38% (28%-48%)*
Q5	10%	15%	6% (-5%-16%)	5%	40%	34% (23%-45%)*
Concentration index (95% CI)	0.28 (0.10-0.46)	-0.01 (-0.11-0.08)		-0.22 (-0.40-0.04)	-0.04 (-0.09-0.00)	
**Gender**						

Male	4%	24%	20% (14%-27%)*	2%	40%	38% (31%-44%)*
Female	6%	20%	14% (9%-19%)*	5%	48%	43% (36%-50%)*
**Ethnicity**						
Scheduled caste	6%	20%	14% (5%-23%)	5%	40%	35% (27%-44%)*
Scheduled tribe	3%	24%	21% (13%-28%)*	-	-	-
Other backward caste	4%	23%	19% (13%-25%)*	2%	48%	46% (40%-52%)*
Other	11%	17%	6% (-5%-19%)	5%	39%	34% (23%-45%)*
**Maternal education**						
Mothers with <1 yr of schooling	5%	21%	16% (9%-22%)*	2%	45%	43% (37%-50%)*
Mothers with ≥1 yr of schooling	4%	23%	19% (13%-24%)*	6%	42%	36% (29%-42%)*
**Antibiotics**						
**Socioeconomic status**						
Q1	11%	34%	23% (13%-34%)*	19%	24%	5% (-5%-14%)
Q2	16%	20%	4% (-6%-15%)	23%	33%	9% (-1%-20%)**
Q3	20%	39%	19% (7%-31%)*	32%	27%	-5% (-18%-7%)
Q4	17%	35%	18% (7%-29%)*	34%	23%	-10% (-23%-3%)
Q5	14%	31%	17% (3%-31%)*	40%	34%	-6% (-22%-9%)
Concentration index (95% CI)	0.09 (-0.02-0.20)	0.02 (-0.05-0.10)		0.06 (-0.00-0.12)	0.07 (0.01-0.12)	
**Gender**						
Male	16%	30%	14% (7%-21%)*	28%	29%	1% (-6%-8%)
Female	16%	35%	19% (12%-26%)*	30%	27%	-4% (-12%-5%)
**Ethnicity**						
Scheduled caste	16%	43%	26% (13%-40%)*	28%	29%	0% (-9%-10%)
Scheduled tribe	16%	32%	16% (7%-25%)*	-	-	-
Other backward caste	12%	26%	14% (6%-22%)*	30%	28%	-2% (-11%-6%)
Other	26%	38%	12% (-7%-32%)	28%	27%	-1% (-15%-13%)
**Maternal education**						
Mothers with <1 yr of schooling	14%	35%	21% (14%-29%)*	24%	26%	2% (-4%-9%)
Mothers with ≥1 yr of schooling	18%	30%	12% (5%-19%)*	35%	30%	-5% (-14%-4%)
**Other treatment: Unknown powder, tablets, syrup, IV fluid**						
**Socioeconomic status**						
Q1	53%	17%	-36% (-48%- -25%)*	70%	45%	-25% (-39%- -12%)*
Q2	52%	23%	-29% (-41%- -17%)*	77%	34%	-44% (-56%- -32%)*

Q3	55%	12%	-44% (-55%- -32%)*	64%	39%	-25% (-37%- -13%)*
Q4	45%	10%	-35% (-46%- -23%)*	60%	44%	-16% (-29%- -2%)*
Q5	53%	12%	-41% (-56%- -25%)*	52%	42%	-10% (-26%-6%)
Concentration index (95% CI)	0.05 (0.01-0.08)	-0.01 (-0.06-0.04)		-0.01 (-0.04-0.02)	0.00 (-0.04-0.05)	
**Gender**						
Male	51%	13%	-37% (-45%- -30%)	66%	43%	-22% (-32%- -13%)*
Female	52%	16%	-36% (-43%- -28%)	65%	37%	-28% (-37%- -19%)*
**Ethnicity**						
Scheduled caste	57%	16%	-41% (-55%- -26%)	63%	41%	-22% (-34%- -10%)*
Scheduled tribe	48%	15%	-34% (-45%- -23%)	-	-	-
Other backward caste	52%	14%	-38% (-46%- -30%)	67%	40%	-27% (-37%- -17%)*
Other	49%	16%	-33% (-50%- -16%)	65%	41%	-24% (-38%- -9%)*
**Maternal education**						
Mothers with <1 yr of schooling	56%	11%	-45% (-52%- -38%)*	71%	44%	-27% (-36%- -19%)*
Mothers with ≥1 yr of schooling	46%	17%	-29% (-37%- -21%)*	58%	37%	-22% (-31%- -12%)*
**Home fluids**						
**Socioeconomic status**						
Q1	24%	29%	5% (-8%-18%)	38%	35%	-4% (-19%-11%)
Q2	20%	28%	8% (-4%-20%)	36%	29%	-7% (-20%-6%)
Q3	16%	28%	12% (1%-23%)	38%	27%	-11% (-24%-2%)**
Q4	21%	37%	16% (4%-28%)	35%	33%	-2% (-16%-11%)
Q5	30%	48%	18% (2%-34%)	40%	30%	-10% (-26%-7%)
Concentration index (95% CI)	0.03 (-0.08-0.14)	0.10 (0.01- 0.19)		0.04 (-0.02-0.10)	0.06 (0.00-0.11)	
**Gender**						
Male	22%	32%	11% (3%-19%)	36%	29%	-7% (-17%-3%)
Female	23%	32%	9% (1%-18%)	39%	32%	-7% (-16%-3%)
**Ethnicity**						
Scheduled caste	18%	31%	13% (1%-26%)	37%	26%	-10% (-22%-1%)**

Scheduled tribe	22%	34%	12% (2%-21%)	-	-	-
Other backward caste	25%	33%	8% (-2%-18%)	37%	31%	-6% (-17%-5%)
Other	19%	27%	8% (-8%-24%)	41%	40%	-1% (-17%-14%)
**Maternal education**						
Mothers with <1 yr of schooling	20%	36%	16% (8%-24%)	35%	27%	-8% (-17%-2%)
Mothers with ≥1 yr of schooling	25%	30%	5% (-3%-13%)	41%	35%	-6% (-16%-5%)
